# Anti-HDGF Antibody Targets EGFR Tyrosine Kinase Inhibitor–Tolerant Cells in NSCLC Patient-Derived Xenografts

**DOI:** 10.1158/2767-9764.CRC-24-0020

**Published:** 2024-09-03

**Authors:** Cindy Q. Zhou, Ariel Li, Kaoru Ri, Ahmed S. Sultan, Hening Ren

**Affiliations:** Department of Oncology and Diagnostic Sciences, University of Maryland School of Dentistry, Baltimore, Maryland.

## Abstract

Constitutively active mutant EGFR is one of the major oncogenic drivers in non–small cell lung cancer (NSCLC). Targeted therapy using EGFR tyrosine kinase inhibitor (TKI) is a first-line option in patients that have metastatic or recurring disease. However, despite the high response rate to TKI, most patients have a partial response, and the disease eventually progresses in 10 to 19 months. It is believed that drug-tolerant cells that survive TKI exposure during the progression-free period facilitate the emergence of acquired resistance. Thus, targeting the drug-tolerant cells could improve the treatment of NSCLC with *EGFR* mutations. We demonstrated here that *EGFR*-mutant patient-derived xenograft tumors responded partially to osimertinib despite near-complete inhibition of EGFR activation. Signaling in AKT/mTOR and MAPK pathways could be reactivated shortly after initial inhibition. As a result, many tumor cells escaped drug killing and regained growth following about 35 days of continuous osimertinib dosing. However, when an antibody to hepatoma-derived growth factor (HDGF) was given concurrently with osimertinib, tumors showed complete or near-complete responses. There was significant prolongation of progression-free survival of tumor-bearing mice as well. IHC and Western blot analysis of tumors collected in the early stages of treatment suggest that increased suppression of the AKT/mTOR and MAPK pathways could be a mechanism that results in enhanced efficacy of osimertinib when it is combined with an anti-HDGF antibody.

**Significance:**

These results suggest that HDGF could be critically involved in promoting tolerance to TKI in patient-derived xenografts of NSCLC tumors. Blocking HDGF signaling could be a potential means to enhance EGFR-targeted therapy of NSCLC that warrants further advanced preclinical and clinical studies.

## Introduction

Lung cancer remains a major disease and is the leading cause of cancer-related death worldwide (1). In non–small cell lung cancer (NSCLC), the major form of lung cancer, the activating mutation of *EGFR* is an important oncogenic driver. The incidence rate of NSCLC with *EGFR* mutation ranges from 10% to 15% in patients of European origin and 30% to 60% in East Asian patient populations (2). Patients with lung cancer with *EGFR* mutation are often nonsmokers, younger than the median age of average patients with lung cancer, and a higher percentage of them are women.

EGFR is a member of the ERBB family receptor tyrosine kinase vital for the survival, growth, migration, and differentiation of epithelial cells (3). Structurally, EGFR consists of an amino-terminal extracellular ligand-binding domain, linked to a transmembrane region, followed by the kinase domain and a carboxyl-terminal-regulatory domain. Engagement of EGFR ligands, such as EGF or amphiregulin, induces receptor dimerization, followed by autophosphorylation of several tyrosine residues in the regulatory domain, leading to the release of inhibition on the kinase and activation of EGFR. The activated kinase in turn recruits and activates several downstream pathways, including Ras-MAPK, PI3K/AKT/mTOR, that are major drivers of survival and proliferation (4). Dysregulated EGFR activation in the absence of proper physiologic signal plays a crucial role in oncogenesis and in maintaining the malignancy of lung cancer and therefore an ideal therapeutic target (5).

Gain-of-function mutation is a common mechanism that can result in abnormal EGFR activation. Among the more than 30 activating mutations in EGFR kinase domain identified in treatment-naïve tumors, exon 19 in-frame deletion and exon 21 L858R mutation account for about 90% of the incidences. Exon 20 in-frame deletion and exon 19 mutations account for about 10% (6). EGFR with exon 19 deletion, L858R, and G719X mutations can be inhibited by several classes of EGFR tyrosine kinase inhibitors (TKI). They are often referred to as the classical sensitizing *EGFR* mutation. Inhibition of their activity in NSCLC cells causes growth arrest and cell death, typically at nanomolar concentrations under cell culture conditions. Clinically approved EGFR inhibitors for NSCLC treatment include the first-generation competitive inhibitors, such as erlotinib and gefitinib, the second-generation irreversible pan-ERBB family inhibitors, such as afatinib and dacomitinib, and the third-generation mutation-specific inhibitors, such as osimertinib and lazertinib. EGFR-targeted therapy has achieved great clinical success. In patients with NSCLC with metastatic or recurring diseases harboring sensitizing *EGFR* mutation, targeted therapy using these small-molecule TKIs resulted in significantly increased progression-free survival (PFS), compared with platinum doublet chemotherapy (10–19 vs. 5.4 months), and extension of overall survival (OS; 31–38 vs. 26 months; refs. 7–12). It has become one of the preferred first-line choices for these patients.

However, despite the high response rate (∼80%) and significantly extended PFS, few patients have complete responses. In most patients, the disease will eventually progress as clones with acquired drug resistance emerge. Extensive studies have identified a complex mechanism of resistance that causes treatment failure (13, 14). About 50% of the resistance to the first or second-generation TKIs occurs because of secondary T790M mutation in *EGFR*. Other mechanisms that confer resistance include amplification of *EGFR* or activation of other receptor tyrosine kinases, such as ERBB2 and MET, mutation of EGFR downstream signaling effectors, and alteration in cell differentiation status, such as small cell lung cancer transformation, epithelial-to-mesenchymal transition, or acquisition of stem cell–like state (15). Mutation in C797S or loss of T790M can result in resistance to osimertinib in addition to other target-independent alterations (16, 17). Many of these secondary alterations bestow acquired resistance by reactivating the major prosurvival/proliferative signaling pathways.

In contrast to the mechanism of resistance, the mechanism that drives the tumor to transition from drug-sensitive to drug-resistant is less understood. In most patients, this transition probably happens during the period of PFS that lasts for months. In cultured cells harboring a sensitizing *EGFR* mutation, most of the cells are eliminated by TKI treatment at physiologically achievable plasma concentration within a few days. Long-term exposure to escalating concentrations of TKI is typically used to induce acquired resistant clones (18–23). Many of these *in vitro* established resistant clones have *MET* amplification, demonstrating MET signaling as an effective mechanism to bypass EGFR blockage. Indeed, cell lines with high-level MET expression, such as Hcc827 or PC-9, are the easiest to induce TKI-resistant clones in cell culture by *MET* gene amplification. Clinically, about 5% to 20% of the tumors in patients relapsed on TKI treatment have *MET* amplification (24–28). This suggests other mechanisms probably exist *in vivo* to promote the survival of tumor cells during the PFS in which steady-state plasma concentration of TKI can be achieved in 1 to 2 weeks.

Hepatoma-derived growth factor (HDGF) is a heparin-binding protein first isolated from the hepatoma cell line Hu-7 conditioned medium base on its ability to stimulate Swiss 3T3 cell proliferation (29). It is distinct from hepatocyte growth factor, the ligand of MET, and there is no report of direct interaction of HDGF with MET. In several tumor cell lines, HDGF has been shown to activate PI3K/Akt signaling through interaction with cell surface receptor (30, 31) or activate MAPK/ERK signaling by enhancing KRAS expression (32). Dysregulated overexpression of HDGF has been identified in multiple solid tumors and is associated with inferior clinic outcome of patients with cancer (33). We have shown in our prior studies that there is a strong association of HDGF overexpression with poor performance in patients with NSCLC. Reducing the expression of HDGF in NSCLC cells by siRNA suppresses the tumorigenicity and malignancy of NSCLC cells (34, 35). Furthermore, anti-HDGF antibody could enhance the chemotherapeutic treatment of NSCLC tumor xenografts (36, 37).

In this study, we investigated the mechanism underlying incomplete response to TKI in EGFR-targeted therapy and the role of HDGF in this process in PDX models of NSCLC harboring *EGFR* mutation.

## Materials and Methods

### Drug and recombinant proteins

Osimertinib methanesulfonate (>99%) was obtained from LC Laboratories. The purification of HDGF and generation of antibody were described previously (36). To produce humanized anti-HDGF antibody, cDNA encoding the variable domain of the murine antibody was amplified and then sequenced, and the complementary determinant regions were grafted onto a human IgG1 framework, cloned into a mammalian expression vector, and then expressed in the Expi293 transient expression system (Invitrogen). Recombinant anti-HDGF antibody H3 was purified by standard affinity chromatography, and endotoxin was removed to <0.02 EU/mg. The binding affinity of the humanized antibody to HDGF was determined by surface plasmon resonance on Biacore 3000 using GST-HDGF as the analyte.

### Expression of recombinant HDGF protein for epitope mapping

The DNA sequence encoding GST-HDGF fusion proteins and a series of C-terminal truncations were cloned into pBiEx-1 (Novagen), expressed in BL21(DE3) cells, fractionated by SDS-PAGE, and blotted and probed with recombinant anti-HDGF antibody H3.

### Generation of *HDGF*-knockout cells

*HDGF* single-guide RNA CRISPR/Cas9-targeting constructs (Applied Biological Materials Inc., Cat#23141111) were packaged in lentivirus and used to transduce HEK293 cells. The sequences targeted were targets 1 to 3: GTC​GCG​ATC​CAA​CCG​GCA​GA; targets 2 to 159: TGG​CCT​CCA​CTC​ACG​TCT​CG; and targets 3 to 195: TCT​CCT​TGG​ATT​CCT​CGT​AA. The transduced cells were selected using puromycin to generate polyclonal (pooled) cell lines.

### Tumor models and dosing

PDX models of NSCLC, TM00219 and TM00199 (Table 1), were obtained from The Jackson Laboratory and propagated in immunodeficient NOD.Cg-*Prkdc*^*scid*^*Il2rg*^*tm1Wjl*^/SzJ mice (38). The initial engraftment by the vendor, designated as P0, was harvested and cryopreserved. Low-passage tumors (P2 to P4) were used to prepare treatment cohorts. Briefly, a fresh tumor was cut into ∼2-mm pieces and implanted into the flank of the recipient animal. Female mice were used for both PDX models to match the sex with the human donors. When the tumor volume reached 250 to 300 mm^3^, the mice were randomized into two arms to receive osimertinib (10 mg/kg *per os* for 5 days a week) or osimertinib plus H3 (13 mg/kg intraperitoneally every 3 days). Tumor sizes were measured using a caliper twice a week. The approximate volume was calculated using the formula *V* = *L* × 2*W* × *H*. The use of animals in this study was approved by the UMB Institutional Animal Care and Use Committee.

**Table 1:** Selected characteristics of the PDX models used in the study. Data were extracted from the database of The Jackson Laboratory

Model	Ploidy	*EGFR* mutation	*EGFR* ^CNV^	*MET* ^CNV^	Tumor type	Treatment naïve?	Donor sex
TM00199	2.7135	L858R	3	−1.829 (P0) −2.498 (P1)	Metastatic	No	F
TM00219	2.2851	T790M, E746_A750del	0.7	0.029	Lymph node metastatic	No	F

Abbreviations: CNV, gene copy number variation; F, female.

### IHC staining and evaluation

Formalin-fixed, paraffin-embedded tumor sections were prepared and stained as described (34). Briefly, duplicate sections were stained with diluted antibody at 4°C overnight and then visualized using Elite ABC kit (VectorLabs) and hematoxylin counterstain. The mounted slides were scanned using Olympus VS120 or Aperio CS2 Pathology Slides Scanner at 40× resolution without adjusting or editing. The staining intensity was semiquantitatively evaluated by two team members and a trained pathologist (AS), in which each tumor section was scored negative or weak (i), moderate (ii), and strong (iii).

### Western blot analysis

Soluble proteins were extracted from frozen tumor using PBS buffer containing 1% Triton X-100 supplemented with protease and phosphatase inhibitors, then separated on 4% to 12% SDS-PAGE gradient gel, blotted to the nitrocellulose membrane, and probed sequentially with 5 to 7 antibodies. The membrane was stripped between staining. Antibody reactivity was detected electrochemiluminescently on film, scanned using CanoScan 9000F, and quantified using ImageJ. Images were not adjusted or edited manually.

### *EGFR* exon 20 sequence analysis

Genomic DNA was purified from pellet after tumor protein extraction with proteinase K digestion and silica membrane binding. A 1,320-bp fragment in *EGFR* gene flanking exon 20 was amplified using high-fidelity PCR and primers in introns 19 to 20 (5′-CAC​AGC​ACA​GAG​AGA​CCA​CT-3′) and introns 20 to 21 (5′-CAA​GGT​AAG​CAA​GCC​AGG​CC-3′) and purified and sequenced using a primer in exon 20 (5′-GAA​GCC​TAC​GTG​ATG​GCC​A-3′).

### Antibodies

Antibodies to the following were obtained from Cell Signaling Technology: phospho-EGFR (Tyr1068; #3777, RRID: AB_2096270 and #2234, RRID: AB_331701), phospho-MEK1/2 (Ser217/221; #9121, RRID: AB_331648), MEK1/2 (#9122, RRID: AB_823567), phospho-p44/42 MAPK (Erk1/2; Thr202/Tyr204; #9101, RRID: AB_331646 and #4307, RRID: AB_2315112), p44/42 MAPK (Erk1/2; #9102, RRID: AB_330744), phospho-Akt (Ser473; #4060, RRID: AB_2315049 and #9271, RRID: AB_329825), Akt (#9272, RRID: AB_329827), phospho-PRAS40 (Thr246; #2997, RRID: AB_2258110), PRAS40 (#2610, RRID: AB_916206), phospho-p70 S6 kinase (Thr389; #9234, RRID: AB_2269803), p70 S6 kinase (#2708, RRID: AB_390722), phospho-4EBP1 (Thr37/46; #2855, RRID: AB_560835), 4EBP1 (#9644, RRID: AB_2097841), Met (#8198, RRID: AB_10858224), and phospho-Met (Tyr1234/1235; #3077, RRID: AB_2143884). Anti-EGFR was obtained from Santa Cruz Biotechnology (Cat#sc-03, RRID: AB_631420). Anti–β-actin was obtained from Sigma (Cat#A1978, RRID: AB_476692). Anti–HDGF T221 was used in previous studies (34, 39).

### Cell lines

HEK293 (CRL-1573) and Hcc827 (CRL-2868) were obtained from ATCC. Expi293F was part of Invitrogen Cat#A14635. The cell lines were expanded upon arrival, and multiple aliquots of low-passage cells (P2–P3) were cryopreserved. Upon recovery, the cells were cultured in media containing tiamulin fumarate, followed by minocycline (Roche, BM-Cyclin). All cells were tested negative for *Mycoplasma* contamination monthly using the MycoFluor Kit (Invitrogen). Cells within passage 6 were used for all experiments.

### Statistical analysis

Differences in PFS were analyzed by Kaplan–Meier survival curves using SRplot (http://www.bioinformatics.com.cn/srplot). Significance was tested using the log-rank test. Quantitative data of IHC and Western blotting analysis were analyzed using the *t* test (one-tailed homoscedastic).

### Data availability

The data generated in this study are available upon request from the corresponding author.

## Results

### Humanized anti-HDGF antibody binds HDGF with high affinity and specificity

**Figure 1:**
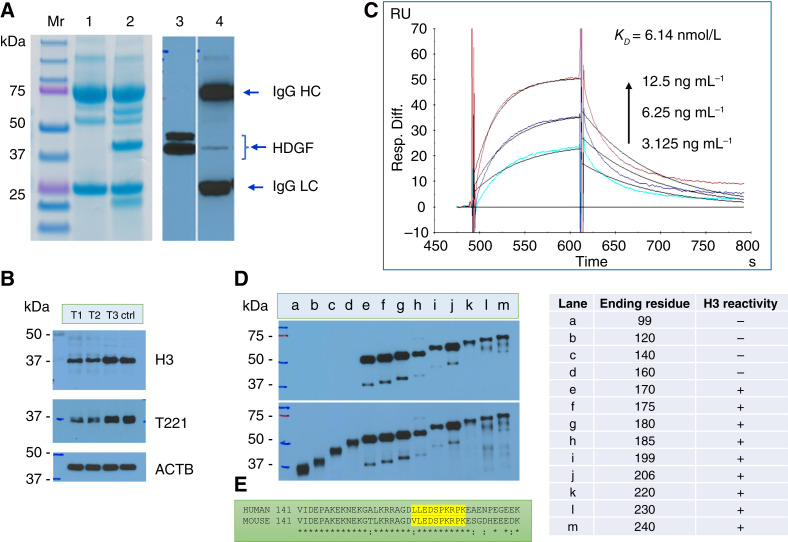
Characteristics of HDGF binding by humanized anti-HDGF antibody H3. **A,** Binding of native HDGF by anti-HDGF antibody H3. Recombinant H3 was immobilized on protein G agarose beads and used to capture HDGF in HEK293 lysate. Lane 1, H3 loaded-beads; lane 2, H3-beads plus HEK292 lysate; lane 3, captured protein probed with rabbit anti–HDGF antibody T221; lane 4, the same blot was striped and probed with goat anti–human IgG antibody. HDGF migrates at 37–42 kDa, larger than the calculated molecular weight of 26.7 kDa. **B,** Specificity of H3 recognition. Western blots of HEK293 parental (ctrl) or pooled HDGF knockout (T1, T2, and T3) cells probed with H3 or T221. The intensity of HDGF staining by H3 or T221 is reduced in the T1 and T2 knockout cells. **C,** Surface plasmon resonance sensorgram of the H3–HDGF interaction. The recombinant antibody was immobilized on the Biacore sensor chip, and GST-HDGF was used as the analyte. **D,** H3 epitope mapping. Bacterial expressing GST-HDGF fusion proteins were lysed, separated by SDS-PAGE, and probed with H3, followed by A2, an antibody that recognizes an epitope in HDGF PWWP domain. The full-length GST-HDGF fusion protein migrated with a molecular weight of 75 kDa (lane m), larger than the predicted size of 52 kDa. The GST-HDGF99 fusion protein (lane a) migrated at the predicted size of 37 kDa. The transition of H3 reactivity happens between constructs ending at residues 160 and 170, indicating that the H3 recognition epitope is in this region. **E,** H3 epitope and flanking sequence in mouse and human HDGF.

The recombinant humanized anti-HDGF antibody H3 binds HDGF in its native form, as shown in its ability to capture HDGF from HEK293 cell lysate (Fig. 1A). The captured HDGF migrated on SDS-PAGE with an apparent molecular weight of about 37 kDa (major) and 42 kDa (minor). The specificity of the H3–HDGF interaction is further validated by co-reduction in the intensity of H3-reactive bands in Western blot of HEK293 cells with CRISPR-Cas9–mediated *HDGF* knockout (Fig. 1B). The H3–HDGF interaction is strong with a dissociation constant (Kd) of 6.14 × 10^–9^ mol/L (6.14 nmol/L) when assayed by surface plasmon resonance using GST-HDGF as the analyte (Fig. 1C). By probing a set of GST-HDGF fusion proteins with sequential C-terminal deletion, the epitope recognized by H3 was mapped to between amino acid residue 160 and 170 in a region predominated by an unfolded sequence (Fig. 1D). This stretch of sequences is highly homologous between the human and mouse (Fig. 1E).

### Incomplete response of PDXs of *EGFR*m+ NSCLC tumors to osimertinib monotherapy

**Figure 2:**
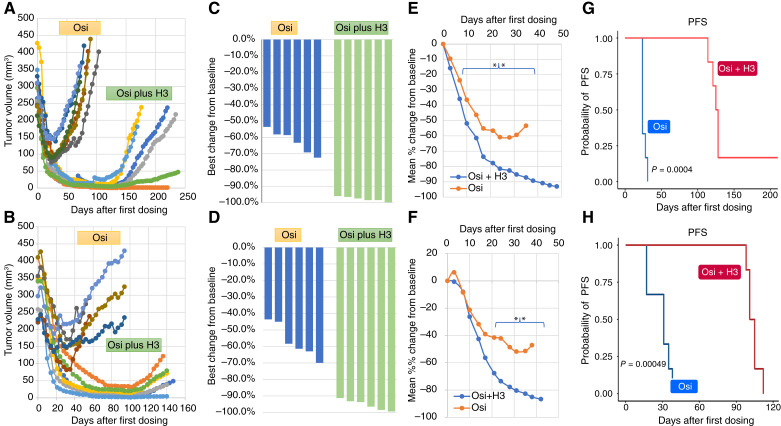
Anti-HDGF antibody enhances the efficacy of EGFR-targeted therapy. Mice with established PDXs of NSCLC tumors were randomized into two arms (*n* = 6 each) to receive osimertinib or osimertinib plus anti-HDGF antibody. **A** and **B,** Spider plot of tumor volume changes in mice treated with osimertinib or osimertinib plus anti-HDGF antibody. **C** and **D,** Waterfall plot of best percent change from baseline of each tumor in the two treatment arms. **E** and **F,** Mean percent change from baseline in the two treatment arms. Asterisks mark the data point in which there is a significant difference between the two treatment arms. **G** and **H,** Kaplan–Meier plot of PFS of mice in the two treatment arms. Graphs show the results of TM00219 (**A**, **C**, **E**, and **G**) and TM00199 (**B**, **D**, **F**, and **H**).

The response of *EGFR*m+ NSCLC tumor to the third-generation EGFR TKI osimertinib was evaluated in established PDX tumors. Continuous osimertinib dosing of mice bearing TM00219 (250–329 mm^3^) or TM00199 (228–410 mm^3^) tumors induced rapid tumor regression (Fig. 2A and B). However, the response was partial with the best percent change from baseline (BCB) ranging from 53% to 72% in TM00219 to 43% to 70% in TM00199 (Fig. 2C and D). In both models, tumor growth resumed after about 24 to 31 days (median, 24 days) for TM00219 or 17 to 38 days (median, 29 days) for TM00199, even with continued osimertinib dosing. Similarly, xenograft tumors derived from Hcc827, an *EGFR*-mutant cell line sensitive to erlotinib *in vitro*, also had incomplete response to erlotinib *in vivo* (Supplementary Fig. S1).

### Enhanced response of PDXs of *EGFR*m+ NSCLC tumors to osimertinib plus anti-HDGF antibody

Next, we examined whether anti-HDGF antibody can alter the tumor response to osimertinib. In established TM00219 tumors (257–427 mm^3^), the humanized anti-HDGF antibody H3 was given at 13 mg/kg concurrently with the start of osimertinib dosing and subsequently maintained at twice per week till tumor relapse or up to 200 days. The combination treatment also induces rapid tumor regression (Fig. 2A) at a pace similar to or slightly faster than that of osimertinib monotherapy as shown in the mean percent change from baseline in tumor volume (Fig. 2E). More importantly, tumor regression proceeded to near completion with the BCB reaching 96% to 99.8% (Fig. 2C). Similarly, in established TM00199 tumors (229–410 mm^3^), osimertinib plus H3 combination also induced rapid tumor regression with BCB ranging from 91% to 99% (Fig. 2B and D) at a pace of tumor regression similar to that of osimertinib monotherapy (Fig. 2F). In both models, there were significant extensions of PFS ranging from 114 to 220 days (median, 128 days) for TM00219 and 98 to 112 days (median, 104 days) for TM00199. The difference in the probability of PFS in the two treatment arms is significant (*P* = 0.0004; Fig. 2G and H). However, H3 alone has no effect on the growth of osimertinib-naïve PDX tumors (Supplementary Fig. S2A and S2B) or tumor progressed on osimertinib monotherapy (Supplementary Fig. S3A and S3B).

### Reactivation of mTOR and MAPK pathways in PDX tumors receiving osimertinib monotherapy

**Figure 3:**
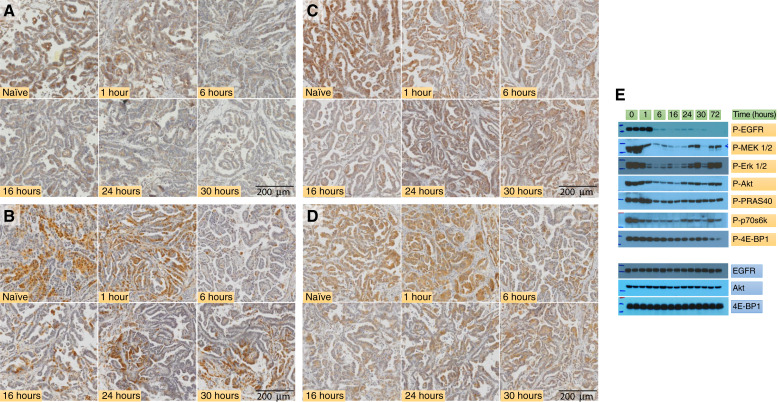
Dynamics of EGFR signaling during the early stage of osimertinib treatment. Mice bearing TM00219 tumors were treated with osimertinib (10 mg/kg *per os*, every 24 hours) for up to 72 hours. Duplicate tumor samples were serially collected at indicated time intervals. Each tumor was split for formalin-fixing and paraffin-embedding and snap-freezing in liquid nitrogen. Duplicate formalin-fixed, paraffin-embedded sections were stained with (**A**) P-EGFR (Y1068), (**B**) P-Erk1/2 (T202/Y204), (**C**) P-Akt1 (S473), and (**D**) P-PRAS40 (T246). Stained slides were scanned using Olympus VS120 Pathology Slide Scanner. Representative images were extracted from OlyVIA at 4× view. Western blots of tumor protein extracts (10 μg/lane) from the corresponding serially collected samples probed with indicated antibodies (**E**).

To identify potential mechanisms leading to the limited response in osimertinib monotherapy, we examined the early changes in the major pro-proliferative signaling pathways downstream of EGFR in serially collected TM00219 tumors after initiation of osimertinib treatment by IHC and Western blotting. After oral osimertinib administration, significant suppression of EGFR Y1068 phosphorylation was observed at 6 hours in IHC-stained tumor tissues (Fig. 3A). In the downstream Akt/mTOR and MAPK pathways, phosphorylation of Erk1/2, Akt, and PRAS40 also showed significant suppression by 16 hours in IHC staining. However, 24 to 30 hours after initiation of osimertinib treatment, there was an apparent increase in phosphorylation of these proteins in IHC-stained tumor samples (Fig. 3B–D). In several tumors, positive phosphoprotein staining was most prominent in small loci. However, at the same time, phosphorylation of EGFR Y1068 remains low (Fig. 3A). Western blot examination of tumor protein extracts from the corresponding serially collected samples confirmed suppression of EGFR Y1068 phosphorylation starting from 6 hours after osimertinib dosing and was undetectable by 72 hours (Fig. 3E). The phosphorylation of MEK1/2 and Erk1/2 of the Ras/MAPK pathway and Akt, PRAS40, and p70S6K of the Akt/mTOR pathway began declining at 6-hour post-osimertinib dosing, remaining low at 16 hours. However, there was a significant increase in phosphorylation of these proteins at 24 hours, in line with the increase in phosphorylation observed in IHC-stained tumor tissues (Fig. 3E). During this period, the total level of these proteins likely remains unchanged as exemplified by EGFR, Akt, and 4EBP1.

### Anti-HDGF antibody attenuates reactivation of Akt/mTOR and MAPK pathways in PDX tumors treated with osimertinib

**Figure 4:**
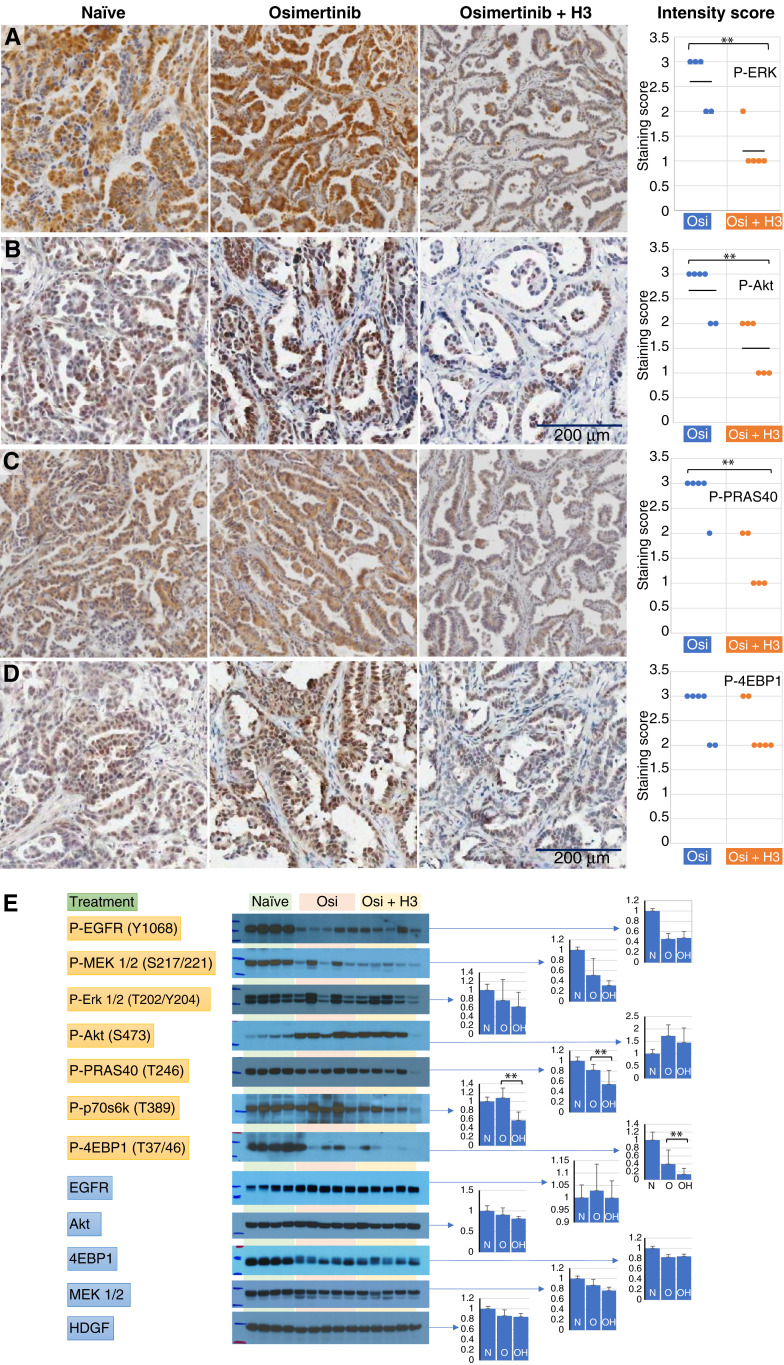
Effect of anti-HDGF antibody on EGFR signaling in osimertinib-treated TM00219 PDX tumor. Mice with established TM00219 PDX tumors were randomized into two arms (*n* = 6 each) to receive osimertinib or osimertinib plus anti-HDGF antibody H3. After 10 days of treatment, tumors were collected from euthanized animals and split for formalin-fixing and paraffin-embedding and Western blot analysis. **A–D,** IHC staining of tumor formalin-fixed, paraffin-embedded sections using (**A**) P-Erk1/2 (T202/Y204), (**B**) P-Akt1 (S473), (**C**) P-PRAS40 (T246), and (**D**) P-4EBP1 (T37/46). Representative fields of stained tumor at 4× view on OlyVIA (P-ERK and P-PRAS40) or 8× view on ImageScope (P-Akt and P-4EBP1) were shown. Tumors staining intensities in the two treatment arms were scored and plotted as the Cleveland dot graph. Naïve tumor staining is also shown for comparison. **E,** Western blots of protein extracts (10 μg/lane) from a matching set of tumors probed with indicated antibodies. The Western blot images were scanned and quantified using ImageJ. Mean density for each treatment group was plotted as a bar graph ± SD, with *Y*-axis showing image density relative to the naïve group. N, naive; O, osimertinib; OH, osimertinib plus anti-HDGF antibody H3. Asterisks mark the datasets in which there is a significant difference between the treatment arms.

We next examined the effect of anti-HDGF antibody on these signaling pathways in tumors treated with osimertinib for 10 to 13 days. At this point of treatment, there was approximately 30% tumor size reduction. In IHC staining of phosphorylated Erk1/2, Akt, PRAS40, and 4EBP1, there were regions of strong positive staining in tumors from the osimertinib monotherapy arm. In contrast, the staining intensities of these phosphorylated proteins in tumors from the osimertinib plus anti-HDGF antibody combination arm were typically much weaker (Fig. 4A–D). The difference in the staining intensity of P-Erk1/2, P-Akt, and P-PRAS40 between the two arms was significant as shown in the Cleveland dot graph.

We further examined the level of phosphorylation in the Akt/TOR and MAPK pathways in protein extracted from these tumors by Western blotting (Fig. 4E). Stronger staining of phosphorylated MEK1/2, Erk1/2, p70S6K, and 4EBP1 was observed in the osimertinib monotherapy arm comparing with the osimertinib plus H3 combination arm, whereas changes in Akt and PRAS40 were not significant. It is interesting to note that the level of EGFR phosphorylation in both treatment arms was increased compared with the P-EGFR level at 72 hours (Fig. 3E). However, the level of P-EGFR in a particular tumor did not always correlate well with the level of phosphorylation in the downstream pathways. In addition, there was a noticeable reduction in the total 4EBP1 level in both treatment arms compared with naïve samples.

### Osimertinib tolerance did not evoke MET activation or *EGFR* C797X secondary mutation

To evaluate the contribution of MET activation in the incomplete response to osimertinib of the PDX tumors, we examined the level of MET and P-MET expression in TM00219 in comparison with Hcc827 cells. In medium containing 10% FBS, Hcc827 cells showed a high level of MET and P-MET expression that can be abolished by treatment with crizotinib. However, the TM00219 PDX tumors displayed significantly lower levels of MET expression and negligible levels of P-MET expression in naïve tumor and in arms treated with osimertinib or osimertinib plus H3 for 13 days. More importantly, the level of MET expression in the PDX tumor did not correlate with the drug treatment regimen (Supplementary Fig. S4A).

We next examined if the C797X secondary mutation is involved in the incomplete response to osimertinib of these PDX tumors. Genomic DNA fragments flanking *EGFR* gene exon 20 were amplified from naïve tumors and tumors treated with osimertinib or osimertinib plus H3 for 13 days and then sequenced. The sequencing chromatograms of the two treated arms were similar to naïve tumors, and no indication of C797X mutation was found (Supplementary Fig. S4B).

### Elevated expression of HDGF in TKI-tolerant tumor cells

**Figure 5:**
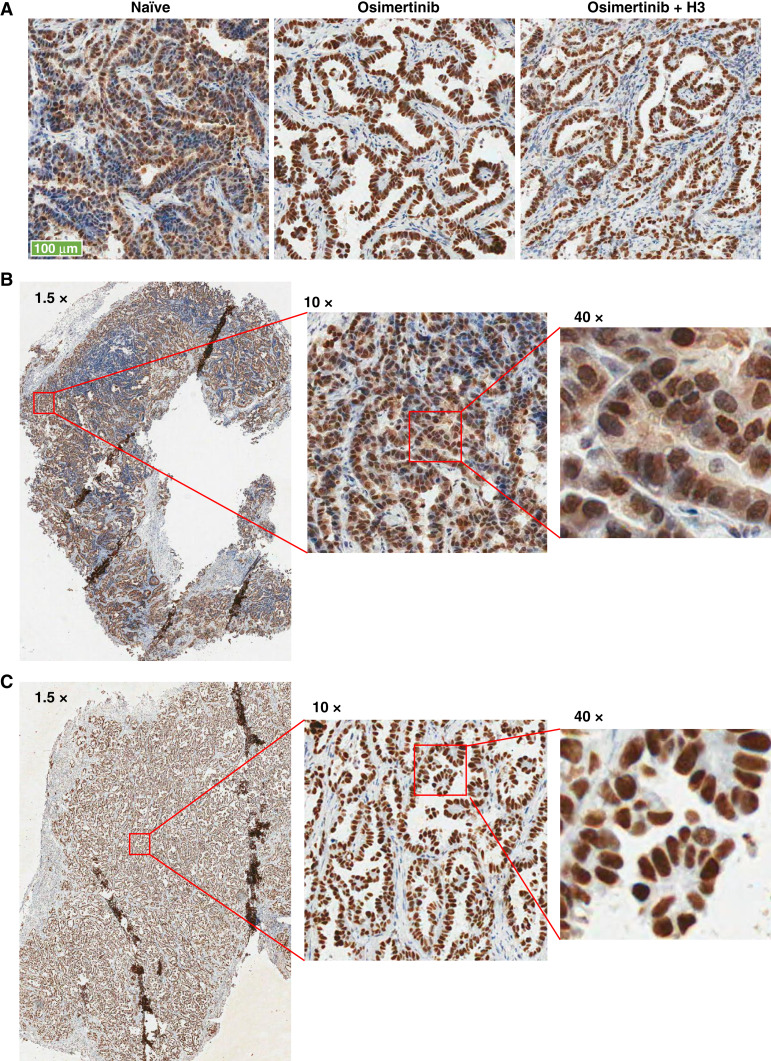
HDGF expression in naïve and osimertinib-treated PDX tumors. Formalin-fixed, paraffin-embedded sections of TM00219 PDX tumors were stained with mouse anti–HDGF antibody T221. Representative fields (10×) of staining in naïve, osimertinib-treated, or osimertinib plus anti-HDGF antibody H3–treated arms (**A**). Representative slides from naïve (**B**) and osimertinib-treated (**C**) tumor viewed under different power: from left to right are low power (1.5×) wide field views, medium power (10×), and high power (40×) view. Images were extracted from ImageScope view.

Next, the relationship of HDGF expression and TKI tolerance was examined in tumors from different treatment arms. In IHC of naïve tumor, HDGF staining was seen in the nucleus of most of the tumor cells with intensity ranging from weak to medium to strong. Weak cytoplasmic staining of HDGF was also observed in naïve tumor cells (Fig. 5A and B). Interestingly, in both osimertinib monotherapy and combination therapy arms, all surviving tumor cells displayed nearly uniform medium strong to strong nuclear staining and negligible cytoplasmic staining (Fig. 5A and C). However, we did not observe significant differences in the mean intensity between osimertinib monotherapy and osimertinib plus H3 combination arms despite some variations within the treatment arms. In addition, we did not observe significant changes in HDGF staining intensity in Western blot analysis of tumor proteins extracted from different treatment arms (Fig. 4E).

## Discussion

EGFR TKIs are the preferred treatment options for patients with advanced NSCLC harboring *EGFR* mutations. However, despite the 10 to 19 months of PFS achieved in many patients, most lesions eventually progress after acquiring resistance to TKI. Furthermore, less than 10% of the patients treated with EGFR TKI achieved a complete response. Most patients experience a partial response with 20% to 80% tumor regression.

In contrast, cultured *EGFR*m+ NSCLC cells lacking concurrent bypassing mutation, such as Hcc827, can be effectively killed within days by TKI at a fraction of the clinically achievable plasma drug concentration, typical submicromolar to nanomolar. However, xenograft tumors derived from Hcc827 showed partial response to TKI (Supplementary Fig. S1). This suggests that *in vivo* environment promotes tumor survival when exposed to TKI. The surviving tumor cells, often referred to as “drug-tolerant persister,” exhibit transient and reversible drug tolerance. Conceivably, the ability of tumor cells to rapidly adapt to the drug-tolerant state upon initiation of TKI administration is crucial for the survival of tumor cells during the early stage of EGFR-targeted therapy. Subsequent evolution and acquisition of resistant drivers within the drug-tolerant cells ultimately give rise to *bona fide* resistant clones, leading to disease progression. The incomplete response and the duration of PFS in many patients undergoing TKI therapy suggest that this drug-tolerance phase may exist prior to overt disease progression.

Recently, there was heightened interest in understanding the mechanisms of drug tolerance (40–42). Activation of RTK bypassing signaling pathways is frequently observed in drug-tolerant persisters due to the highly convergent nature of RTK signaling, such as in other ERBB family of kinases, MET and AXL (43). Co-targeting these kinases is a rational approach to break tumor cell tolerance, either by using inhibitors with a wilder specificity (e.g., afatinib) or a combination of inhibitors (e.g., EGFR TKI plus crizotinib). However, the success of these strategies varies. Afatinib, a pan-ERBB inhibitor, exhibits a similar efficacy profile to the first-generation TKIs (44). Dacomitinib, another pan-ERBB inhibitor, had shown extended PFS and overall survival (45), whereas amivantamab, a bispecific antibody targeting EGFR and MET, has shown improved efficacy in combination with lazertinib as a first-line treatment of *EGFR*m+ NSCLC (46). These results underscore that complex mechanisms may exist to promote tumor cell survival and drive the transition from TKI sensitive to resistant.

In this study, we investigated the role of HDGF in promoting tumor tolerance to osimertinib using two PDX tumor models of NSCLC: TM00199 and TM00219. These models were derived from patients who had progressed on single-agent or combination erlotinib treatment (38).

We observed that the PDX tumors are susceptible to EGFR inhibition. Oral osimertinib administration rapidly and profoundly inhibited phosphorylation at EGFR Y1068 and downstream AKT/MTOR and MAPK pathways. These pathways play a critical role in cell survival and are often associated with secondary mutations, leading to resistance against various anticancer therapies. The swift inhibition of these pathways upon osimertinib administration explained the rapid tumor shrinkage.

However, the response of the PDX tumors to osimertinib was incomplete, with maximum regression ranging from 50% to 70% that achieved around 30 days after treatment initiation, followed by progression. Notably, even at the early stage of treatment with approximately 30% tumor shrinkage, significant recovery of phosphorylation in AKT/MTOR and MAPK pathways was observed. Serially collected samples revealed that near-maximum inhibition of EGFR Y1068 and downstream phosphorylation occurred as early as 6 hours, but subsequent recovery of phosphorylation could be detected within 24 hours. This rapid transition from initial suppression to partial recovery suggests that factors other than drug penetration or novel mutations likely contribute to this phenomenon. Reactivation of EGFR downstream pathways was also observed in other studies using patient-derived tumors (38, 47).

Surprisingly, concurrent administration of an anti-HDGF antibody at the start of osimertinib treatment led to tumor regression exceeding 98%. On average, the progression-free time was four to five times longer than osimertinib monotherapy. This suggests that most persistent tumor cells prior to progression were just transiently tolerant to TKI, as opposing to stable resistance. Their tolerance can be overcome by blocking HDGF.

Analysis of tumors harvested ∼10 days after treatment initiation revealed significantly lower levels of activating phosphorylation in AKT/MTOR and MAPK pathways in the combination arms compared with osimertinib monotherapy. This suggests that HDGF participates in reactivating the prosurvival pathways. The anti-HDGF antibody attenuated this reactivation during osimertinib treatment, enhancing overall efficacy. However, when administrated alone, anti-HDGF antibody did not affect the growth of osimertinib-naïve or post-progression tumors, suggesting that HDGF signaling is relatively weak compared with the dominant oncogenic driver.

HDGF, identified as a mitogen for several cell types (39, 48), contains two nuclear localization signals. In most cell types, HDGF is predominantly localized to the nucleus, where it likely functions as a transcription factor or participates in RNA biogenesis. In this study, negative to strong nuclear staining and weak cytoplasmic staining were observed in treatment-naïve tumor cells. In osimertinib-treated tumors, however, all the surviving tumor cells showed strong nuclear staining, suggesting an increase in the level of HDGF expression. Corroborating with our study, biopsy samples of patients with NSCLC treated with EGFR TKI demonstrated a considerable increase in HDGF staining intensity after relapse (49). Furthermore, HDGF expression in cultured cells with *EGFR* mutation and in xenograft tumors derived from them significantly increased after gefitinib or osimertinib treatment (49). These findings suggest that higher levels of HDGF expression are associated with increased TKI tolerance. As HDGF could promote the expansion of hematopoietic or cancer stem cells, it is possible that tumor cells with higher HDGF expression exhibit more stem cell–like features and are thus more tolerant to TKI. This is particularly interesting considering that chemotherapy could extend the benefit of osimertinib (50), and stem-like cancer cells tend to be more resistant to chemotherapy (51). Targeting HDGF could potentially suppress resistance to chemotherapy too and further enhance the benefit of osimertinib plus chemotherapy. In a previous study, the administration of an anti-HDGF antibody resulted in reduced stem cell features in tumors and enhanced the efficacy of combination chemotherapy in NSCLC tumors (37).

As a protein without a classical secretion signal, HDGF is readily detected in media of cultured NSCLC cells (36). The medium level of HDGF in abnormal fibroblast culture is also elevated (52). It is not entirely clear how HDGF enters the extracellular media. One study suggests that HDGF N-terminal sequence is involved in secretion (53). Stress or apoptosis of cells could increase the level of HDGF released into the media, a condition that is relevant to cancer therapy (54). In patients with NSCLC treated with gefitinib or osimertinib, there were statistically significant increases in plasma concentrations of HDGF (49). Exogenous HDGF could bind to cell surface–displayed nucleolin and activate MAPK/Erk and PI3K/AKT pathways (30). In cultured NSCLC cells or xenograft tumor, the expression of HDGF is positively correlated with the activation of these pathways and the resistance to EGFR TKI; knocking down HDGF diminishes the activation of these pathways and sensitizes the cells to TKI inhibition (49).

Besides the malignant cells, the tumor microenvironment also contains various nonmalignant cells, including vasculature cells, mesenchymal-derived cells such as fibroblast, and immune cells in immune-competent animals and human patients. These cells interact with each other through secreted growth factors, cytokines, or surface-displayed ligands and their receptors. HDGF released by tumor cells could signal to the nonmalignant cells in the tumor microenvironment to promote the survival and malignancy of tumor cells.

Although HDGF-dependent osimertinib-tolerant cells constitute 20% to 30% of the mass in tumors of the osimertinib arm prior to progression, analysis of TM00219 tumors collected approximately 13 days after treatment initiation revealed a low level of MET expression compared with Hcc827 and an insignificant level of P-MET expression. Notably, there were no meaningful differences in the expression of MET and P-MET between osimertinib-naïve and -treated tumors. Furthermore, no secondary mutations at C797 were detected after sequencing *EGFR* exon 20 in these tumors. These data suggest that *MET* amplification/activation and *EGFR* C797X secondary mutation, which are commonly associated with acquired resistance to osimertinib in post-progression tumor in human patients, were unlikely the primary mechanism of osimertinib tolerance at the early stage of osimertinib treatment.

**Figure 6:**
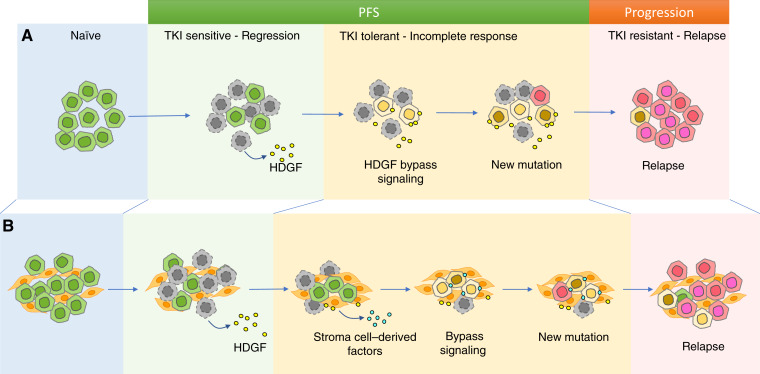
Hypothetic mechanisms of HDGF-mediated tolerance to osimertinib in NSCLC cells with EGFR mutation. In the HDGF-mediated transition model of acquiring resistance to TKI, sensitive cells acquire transitory tolerance to TKI prior to the emergence of new genetic or epigenetic driver alterations. The progression-free period is likely dominated by cells tolerant to TKI. **A,** In the direct mode, HDGF released from dying or dead tumor cells could bind to receptors on surviving tumor cells to enhance their tolerance to osimertinib. **B,** In the indirect mode, HDGF first activates tumor stroma cells, leading to enhanced secretion of stroma cell factors, such as cytokines, growth factors, extracellular vesicle, or expression of the stroma cell surface ligand. These factors then interact with tumor cells, promoting their tolerance to osimertinib. Importantly, these two modes of actions are not mutually exclusive.

Taken together, our data demonstrate that during the early stage of EGFR-targeted therapy, tumor cells can rapidly develop tolerance to osimertinib without acquiring new resistant mutations or activating MET. This process likely involves both tumor cells and nonmalignant cells in the tumor microenvironment (Fig. 6). Subsequent emergence of tumor cells harboring new drug-resistant alterations from the drug-tolerant cells ultimately leads to disease progression while continuing osimertinib treatment. The drug-tolerant cells serve as a vital link in the transition of tumor from a sensitive state to a resistant one. In this intricate process, HDGF plays a crucial role by inducing tumor tolerance, partly through the activation of MAPK/ERK and PI3K/AKT pathways. Blocking HDGF promotes tumor regression and significantly extends survival in tumor-bearing mice.

The current study also showed the complexity of tumor cell signaling under TKI inhibition. For instance, in the first 72 hours of osimertinib treatment, there was near-complete inhibition of EGFR Y1068 phosphorylation. However, rephosphorylation at Y1068 was observed on 10 to 13 days in tumors that had about 30% tumor shrinkage. Surprisingly, this increase in Y1068 phosphorylation did not consistently correlate with downstream activation in the MAPK/ERK and PI3K/AKT pathways. This phenomenon may be explained by cross-talk between EGFR and other kinases. Furthermore, anti-HDGF antibody could not fully prevent the emergence of osimertinib resistance, suggesting that additional bypass pathways may coexist.

### Conclusion

Our study demonstrates that tumor tolerance to TKIs could be a crucial mechanism contributing to incomplete responses during the early phase of EGFR-targeted therapy. HDGF plays a critical role in this process. Concurrent inhibition of HDGF with the initiation of TKI treatment can re-sensitize drug-tolerant cells to TKI, resulting in enhanced tumor regression and delayed disease progression. These findings suggest that targeting drug-tolerant cells early in the treatment process could be a promising strategy for improving EGFR-targeted therapy in NSCLC. Nevertheless, further advanced investigations are necessary to fully elucidate the HDGF-mediated signaling pathway and determine the clinical utility of anti-HDGF antibodies in NSCLC treatment.

## Supplementary Material

Supplement Figure 1 Supplement Figure 1 shows the incomplete response of Hcc827 tumor to erlotinib treatment.

Supplement Figure 2 Supplement Figure 2 shows the Effect of anti-HDGF antibody H3 on treatment naive tumor.

Supplement Figure 3 Supplement Figure 3 shows the Effect of anti-HDGF antibody H3 on post-progression tumor.

Supplement Figure 4 Supplement Figure 4A shows the expression of MET and P-MET in naive and treated tumors. Supplement Figure 4B shows the sequencing result of EGFR exon 20 flanking C797 in naive and treated tumors.
