# Supplementary material for: Anti-HDGF Antibody Targets EGFR Tyrosine Kinase Inhibitor–Tolerant Cells in NSCLC Patient-Derived Xenografts
Source: Cancer Res Commun. 2024 Sep 3;4(9):2308–19. doi: 10.1158/2767-9764.CRC-24-0020 (PMC11370239; doi:10.1158/2767-9764.CRC-24-0020)
Supplement: Supplement Figure 1 — shows the incomplete response of Hcc827 tumor to erlotinib treatment. [file crc-24-0020_supplement_figure_1_suppsf1.pptx]

## Slide 1
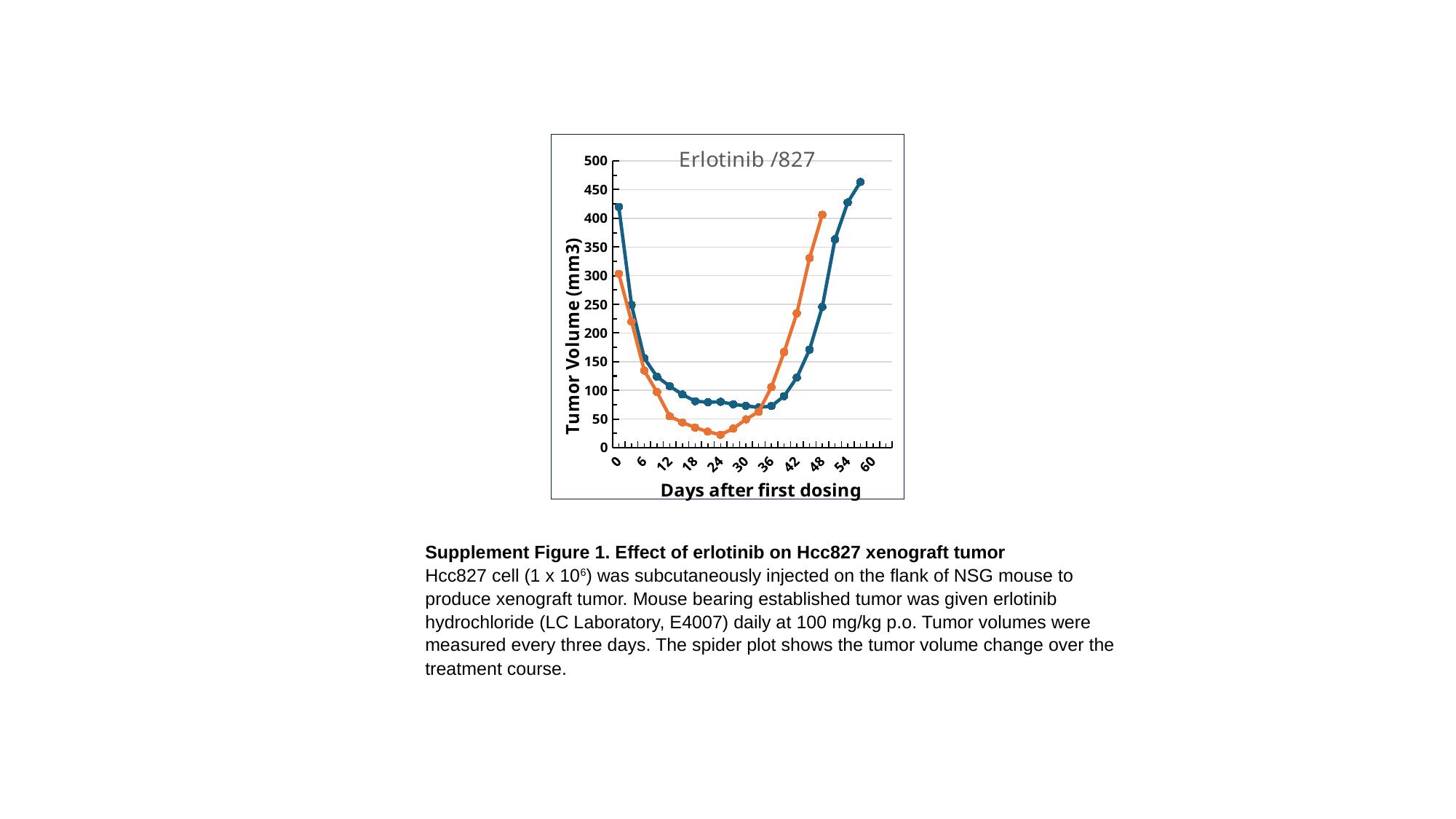

### Chart: Erlotinib /827
| Category | #3L | #6L | |
|---|---|---|---|
| 0 | 419.83 | 303.26 | None |
| 3 | 248.83 | 219.96 | None |
| 6 | 156.28 | 134.56 | None |
| 9 | 123.87 | 96.91 | None |
| 12 | 107.39 | 54.68 | None |
| 15 | 92.7 | 44.11 | None |
| 18 | 81.12 | 34.99 | None |
| 21 | 79.33 | 28.0 | None |
| 24 | 80.0 | 22.53 | None |
| 27 | 75.63 | 33.21 | None |
| 30 | 72.9 | 49.39 | None |
| 33 | 70.27 | 62.78 | None |
| 36 | 72.58 | 105.46 | None |
| 39 | 89.89 | 166.7 | None |
| 42 | 122.3 | 234.41 | None |
| 45 | 171.06 | 330.67 | None |
| 48 | 245.48 | 406.27 | None |
| 51 | 363.38 | None | None |
| 54 | 427.73 | None | None |
| 57 | 463.38 | None | None |
| 60 | None | None | None |
| 63 | None | None | None |Supplement Figure 1. Effect of erlotinib on Hcc827 xenograft tumor
Hcc827 cell (1 x 10^6^) was subcutaneously injected on the flank of NSG mouse to produce xenograft tumor. Mouse bearing established tumor was given erlotinib hydrochloride (LC Laboratory, E4007) daily at 100 mg/kg p.o. Tumor volumes were measured every three days. The spider plot shows the tumor volume change over the treatment course.
