# Supplementary material for: Anti-HDGF Antibody Targets EGFR Tyrosine Kinase Inhibitor–Tolerant Cells in NSCLC Patient-Derived Xenografts
Source: Cancer Res Commun. 2024 Sep 3;4(9):2308–19. doi: 10.1158/2767-9764.CRC-24-0020 (PMC11370239; doi:10.1158/2767-9764.CRC-24-0020)
Supplement: Supplement Figure 2 — shows the Effect of anti-HDGF antibody H3 on treatment naive tumor. [file crc-24-0020_supplement_figure_2_suppsf2.pptx]

## Slide 1
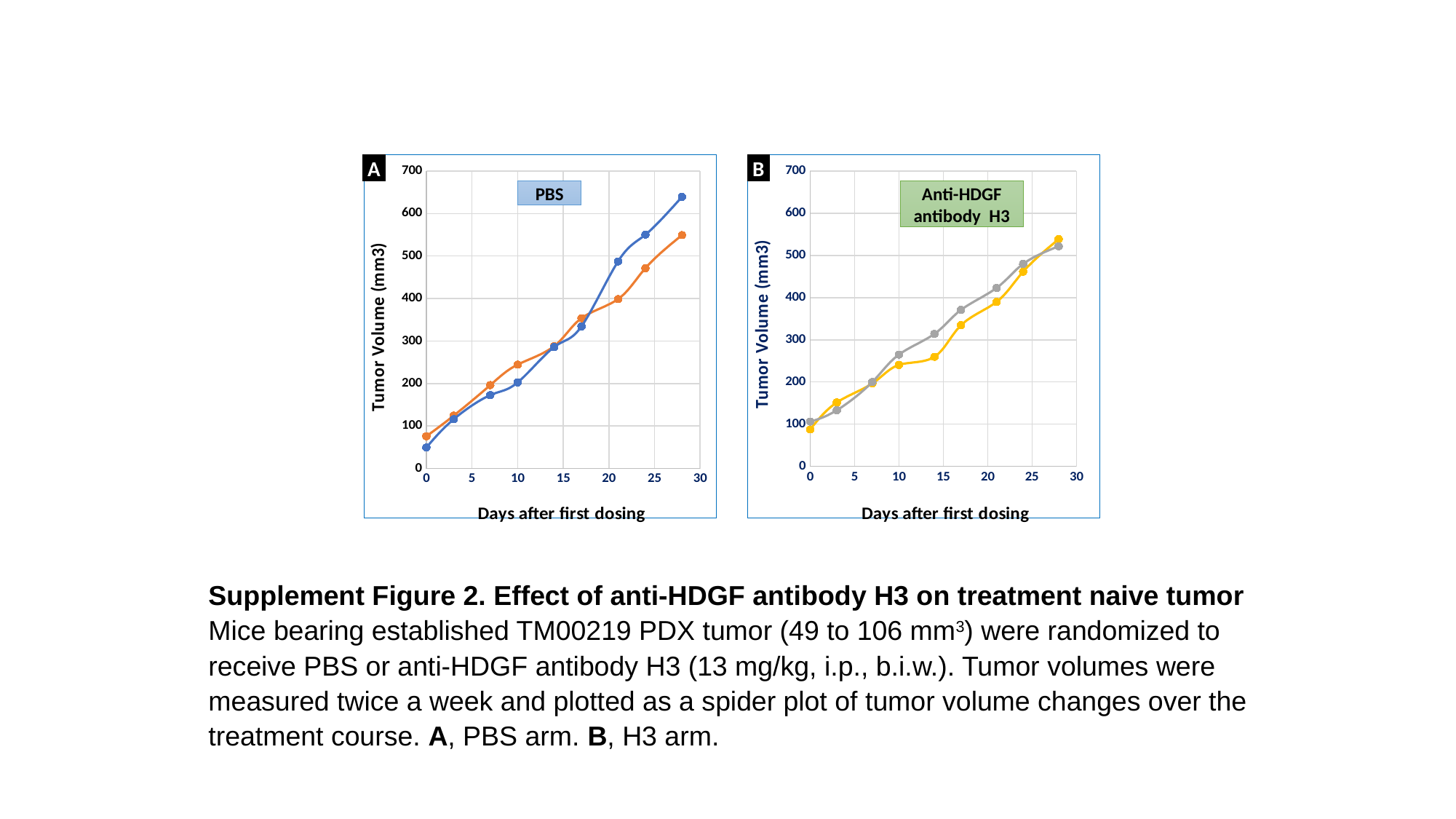

A
### Chart
| Category | 14L | 15L |
|---|---|---|
### Chart
| Category | 30L | 30R |
|---|---|---| B
PBS
Anti-HDGF antibody H3
Supplement Figure 2. Effect of anti-HDGF antibody H3 on treatment naive tumor
Mice bearing established TM00219 PDX tumor (49 to 106 mm3) were randomized to receive PBS or anti-HDGF antibody H3 (13 mg/kg, i.p., b.i.w.). Tumor volumes were measured twice a week and plotted as a spider plot of tumor volume changes over the treatment course. A, PBS arm. B, H3 arm.
