# Supplementary material for: Anti-HDGF Antibody Targets EGFR Tyrosine Kinase Inhibitor–Tolerant Cells in NSCLC Patient-Derived Xenografts
Source: Cancer Res Commun. 2024 Sep 3;4(9):2308–19. doi: 10.1158/2767-9764.CRC-24-0020 (PMC11370239; doi:10.1158/2767-9764.CRC-24-0020)
Supplement: Supplement Figure 3 — shows the Effect of anti-HDGF antibody H3 on post-progression tumor. [file crc-24-0020_supplement_figure_3_suppsf3.pptx]

## Slide 1
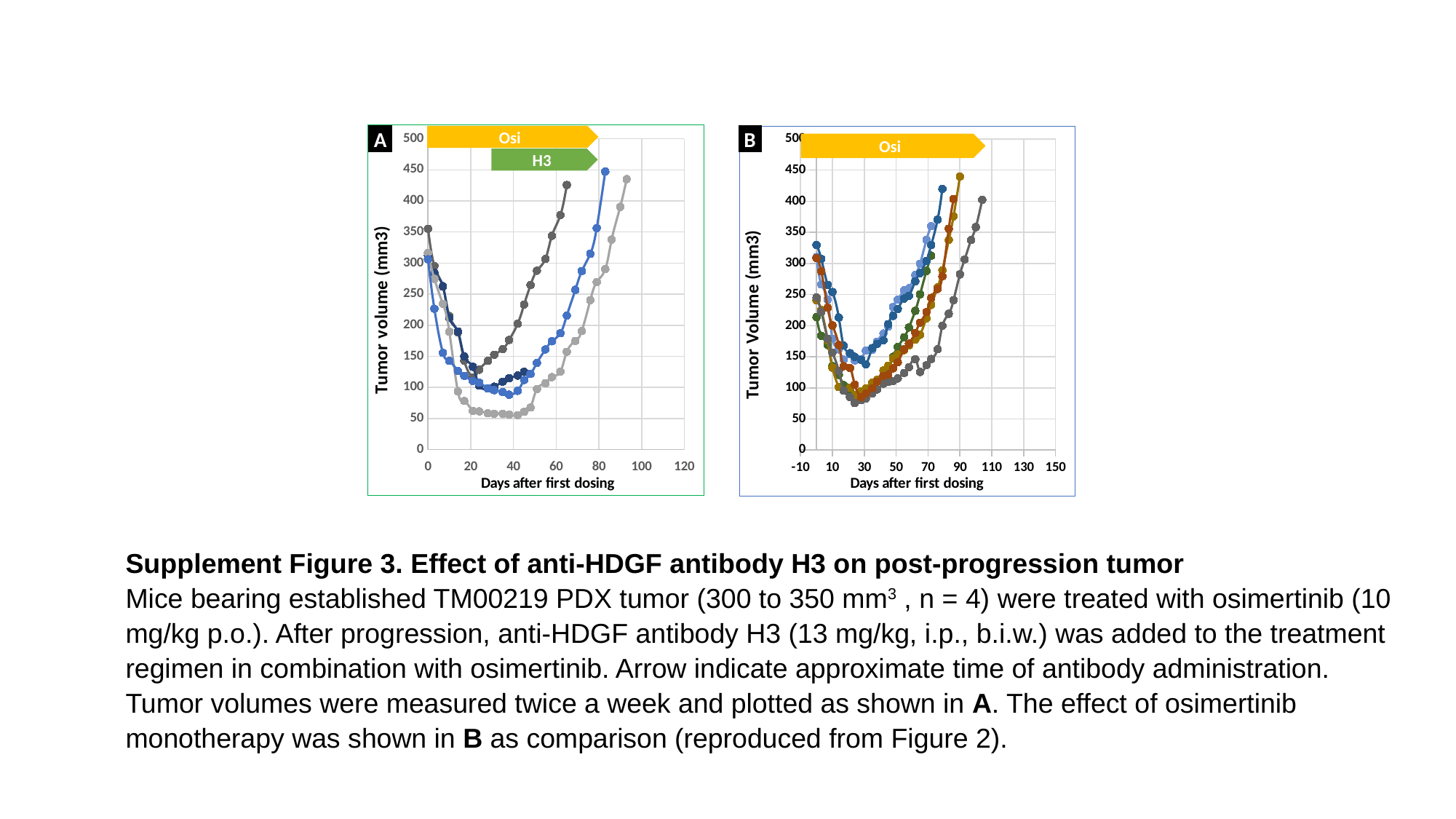

### Chart
| Category | HGF #14 L (F) | HGF #15 L (F) | HGF #31 L (F) | HGF #32 L (F) |
|---|---|---|---|---|Osi
 A
### Chart
| Category | | | | | | |
|---|---|---|---|---|---|---| B
Osi
H3
Supplement Figure 3. Effect of anti-HDGF antibody H3 on post-progression tumor
Mice bearing established TM00219 PDX tumor (300 to 350 mm3 , n = 4) were treated with osimertinib (10 mg/kg p.o.). After progression, anti-HDGF antibody H3 (13 mg/kg, i.p., b.i.w.) was added to the treatment regimen in combination with osimertinib. Arrow indicate approximate time of antibody administration. Tumor volumes were measured twice a week and plotted as shown in A. The effect of osimertinib monotherapy was shown in B as comparison (reproduced from Figure 2).
