# Supplementary material for: Anti-HDGF Antibody Targets EGFR Tyrosine Kinase Inhibitor–Tolerant Cells in NSCLC Patient-Derived Xenografts
Source: Cancer Res Commun. 2024 Sep 3;4(9):2308–19. doi: 10.1158/2767-9764.CRC-24-0020 (PMC11370239; doi:10.1158/2767-9764.CRC-24-0020)
Supplement: Supplement Figure 4 — A shows the expression of MET and P-MET in naive and treated tumors. Supplement Figure 4B shows the sequencing result of EGFR exon 20 flanking C797 in naive and treated tumors. [file crc-24-0020_supplement_figure_4_suppsf4.pptx]

## Slide 1
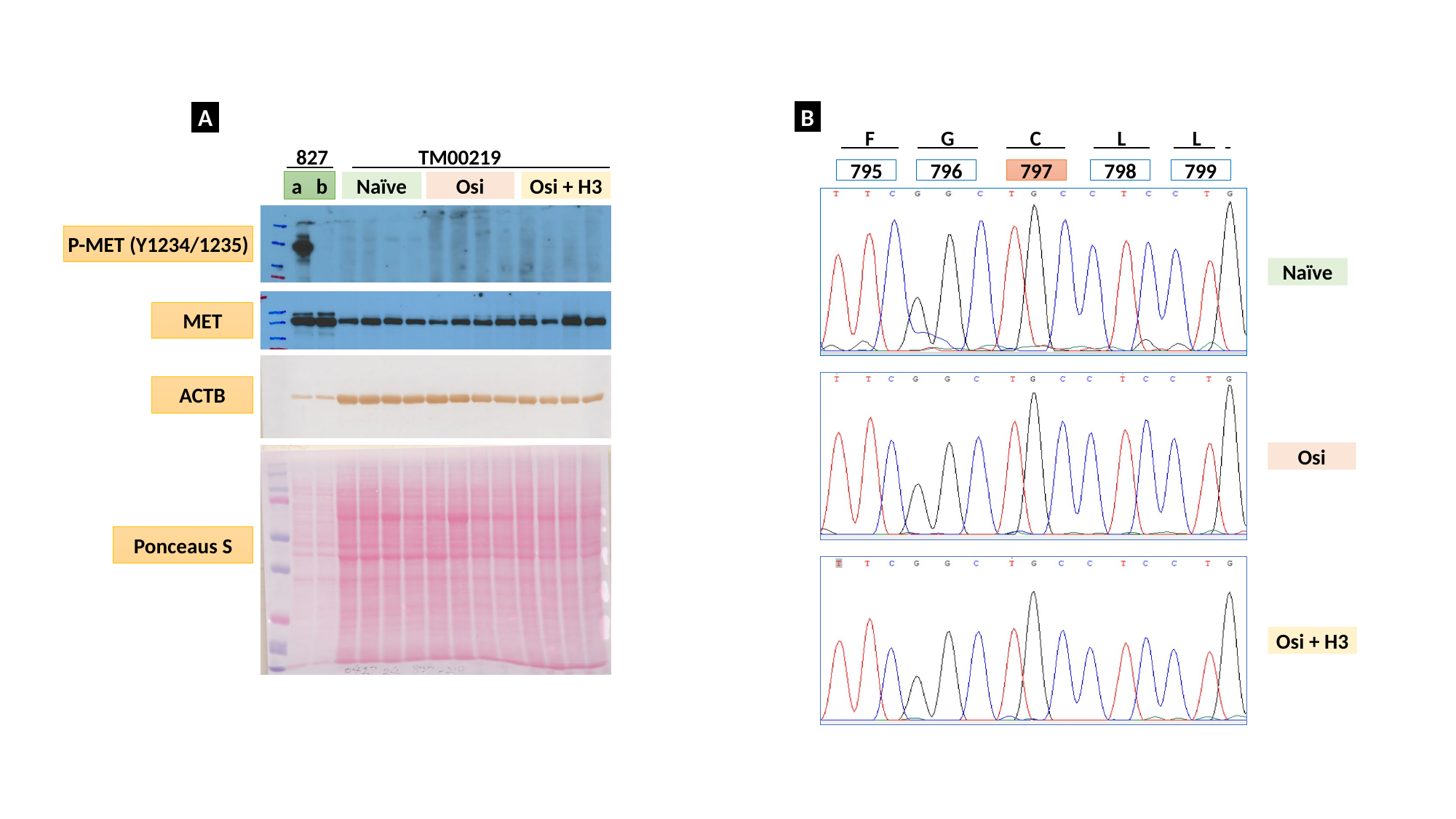

B
 A
 F G C L L l
 827
 TM00219
795
796
797
798
799
a b
Naïve
Osi
Osi + H3
P-MET (Y1234/1235)
Naïve
MET
ACTB
Osi
Ponceaus S
Osi + H3

## Slide 2
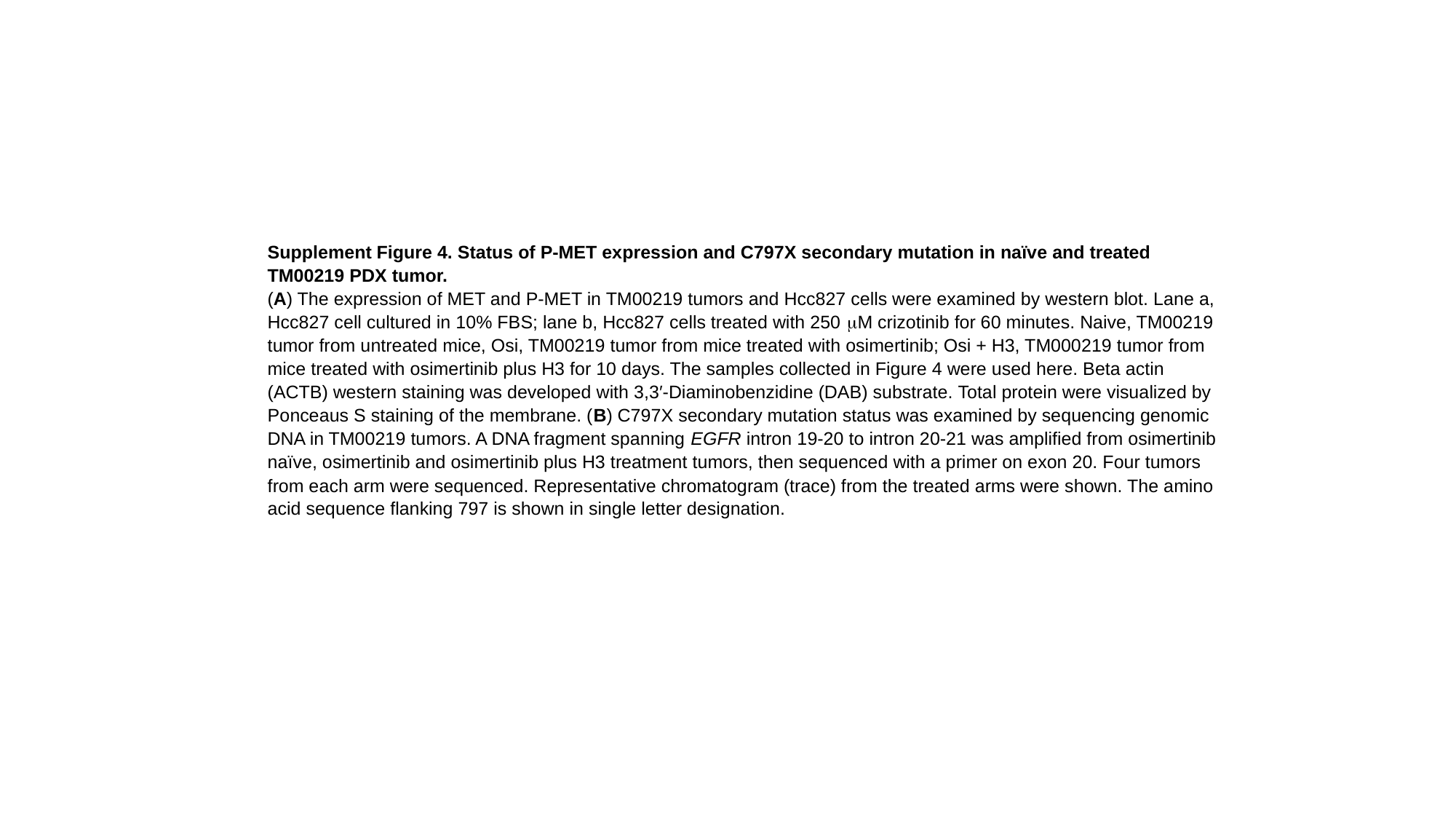

Supplement Figure 4. Status of P-MET expression and C797X secondary mutation in naïve and treated TM00219 PDX tumor.
(A) The expression of MET and P-MET in TM00219 tumors and Hcc827 cells were examined by western blot. Lane a, Hcc827 cell cultured in 10% FBS; lane b, Hcc827 cells treated with 250 μM crizotinib for 60 minutes. Naive, TM00219 tumor from untreated mice, Osi, TM00219 tumor from mice treated with osimertinib; Osi + H3, TM000219 tumor from mice treated with osimertinib plus H3 for 10 days. The samples collected in Figure 4 were used here. Beta actin (ACTB) western staining was developed with 3,3′-Diaminobenzidine (DAB) substrate. Total protein were visualized by Ponceaus S staining of the membrane. (B) C797X secondary mutation status was examined by sequencing genomic DNA in TM00219 tumors. A DNA fragment spanning EGFR intron 19-20 to intron 20-21 was amplified from osimertinib naïve, osimertinib and osimertinib plus H3 treatment tumors, then sequenced with a primer on exon 20. Four tumors from each arm were sequenced. Representative chromatogram (trace) from the treated arms were shown. The amino acid sequence flanking 797 is shown in single letter designation.
